# Enhancing monitoring of rewilding progress through wildlife tracking and remote sensing

**DOI:** 10.1371/journal.pone.0253148

**Published:** 2021-07-09

**Authors:** Julia Carolina Mata, Robert Buitenwerf, Jens-Christian Svenning

**Affiliations:** 1 Department of Biology, Center for Biodiversity Dynamics in a Changing World (BIOCHANGE), Aarhus University, Aarhus, Denmark; 2 Department of Biology, Section for Ecoinformatics and Biodiversity, Aarhus University, Aarhus, Denmark; Qinghai University, CHINA

## Abstract

Defaunation is a global threat to biodiversity that can be counteracted through trophic rewilding, a restoration strategy that promotes self-regulating ecosystems through active reintroductions or passive management. In order to estimate success in restoration initiatives, progress of the rewilding projects is measured and monitored. However, a spatially explicit understanding of rewilding and rewilding potential in a rewilding site has been absent so far. We present a novel approach for monitoring rewilding progress that focuses on a spatially explicit estimate of progress and ecological integrity within rewilding initiatives. This framework uses habitat classification of the site and tracking data of the reintroduced animals, to model their habitat selection. Through this we measure and map realized and potential rewilding. We operationalize the framework in an ongoing rewilding project in the Iberá Wetlands, Corrientes, Argentina. The majority of areas (76%) predicted to be occupied by reintroduced fauna were only predicted to be selected by one species. Of the four species in the rewilding project, only the giant anteater (*Myrmecophaga tridactyla*) filled the majority of its potential distribution, whereas pampas deer (*Ozotoceros bezoarticus*), collared peccary (*Pecari tajacu*) and lowland tapir (*Tapirus terrestris*) filled less than 23% of theirs. After rewilding we found a 10% increase in the proportion of the study area with high ecological integrity. Through this case study, we showed that this framework can be used to assess the spatial progress of a rewilding site. By incorporating wildlife tracking and satellite-based remote sensing, we are integrating a spatial component to monitoring of rewilding projects that should lead to more detailed understanding of the progress of rewilding. Applying this framework would facilitate decision-making for practitioners and inform species management plans.

## Introduction

Defaunation—defined as the loss or decline of animal species from ecological communities–is a crucial component of biodiversity loss [1, 2]. The loss of fauna has negative consequences on phylogenetic and functional diversity, ecosystem functions and services, and has cascading effects on other taxa [1, 3, 4]. Habitat and species conservation initiatives aim to stop defaunation, but active faunal restoration can also be implemented to overcome persistent community simplification due to prior defaunation [2, 4].

Rewilding can be defined as a restoration strategy to promote self-regulating ecosystems through restoring ecological factors and processes while reducing human pressures [5, 6]. Many rewilding initiatives conform with the concept of trophic rewilding, the use of species reintroductions to restore top-down interactions and promote self-regulating ecosystems [7]. Rewilding is applied to restore ecosystem functionality such as predation, herbivory, physical disturbance of vegetation, nutrient distribution, and fire dynamics [3, 4, 8, 9]. There is growing interest in rewilding from policy and rising empirical evidence on its effects [10–12].

A well designed monitoring and evaluation system is fundamental to assess progress towards conservation aims [13]. Conservation monitoring approaches have existed for more than a century and include a wide range of sampling methods and evaluations [13, 14]. Concept development studies on rewilding have highlighted the importance of monitoring not only for evaluating the success of rewilding projects but also to gain knowledge on how to best implement this restoration strategy [7, 15]. Monitoring rewilding progress requires technical and funding resources that many projects do not have. Significant effects of rewilding on the ecosystem may take several years to emerge, necessitating substantial funding to cover this timeframe [16]. Rewilding projects are by definition generally open-ended, without a fixed end state, which adds complexities to defining and monitoring their success [15, 17] and may add perceived uncertainty [18]. Monitoring frameworks specific for measuring rewilding progress have been developed recently [19, 20]. They allow practitioners to assess qualitative progress such as: ecological processes comprising trophic complexity, disturbance and dispersal [19], as key aspects of ecological integrity, and also human control and pressures [20]. Inspired by these monitoring frameworks, we aim to provide a more quantitative implementation to assess rewilding progress through monitoring of the spatial reversal of defaunation with the use of wildlife tracking methods and remote sensing, and thus assess the extent to which the rewilding initiatives achieve the expected restoration of ecosystem functions.

Spatial data on ecosystems and habitats can be supplied by remote sensing. Remote sensing has been used extensively for conservation monitoring regarding alterations in species diversity and distribution, changes in habitat area, habitat degradation, and temporal or spatial trends in pressures and threats [21]. It has also been used in rewilding science to identify possible rewilding sites [22, 23], and to study the habitat selection and species range size of reintroduced fauna [24–26].

Without quantitative means to monitor and evaluate progress, rewilding projects will lack the tools to oversee and learn from their development [27, 28]. We developed a monitoring framework that provides a spatially explicit estimate of progress and ecological integrity within rewilding initiatives. Through the use of spatial wildlife tracking data and high resolution satellite images, we incorporated tools already used in rewilding science: habitat selection and species range analyses. We applied and tested the framework in a rewilding project in a South American forest-savanna-grassland mosaic that is being actively rewilded through species reintroductions [29]. South America has lost more than 70% of its megafauna species during the late Quaternary extinction [8, 30–32] and has been further defaunated since European colonization [3, 29]. We propose the incorporation of remote sensing and spatial analysis into rewilding monitoring to assess refaunation dynamics, thus integrating a spatial and quantitative component through which to spatially measure rewilding progress and potential within a site.

## Methods

### Study site

The Iberá wetland is a large subtropical wetland ecosystem in north-eastern Argentina, of which we focus on the terrestrial ecosystems [33, 34]. The weather is humid subtropical with heavier rainfall in spring and summer, a dryer season during fall and winter, and a mean annual precipitation of 1400 mm. Average temperatures are 27 ˚C in summer and 16 ˚C in winter [34–36]. This region is comprised of a mosaic of marshes, swamps, grasslands, savannas and gallery forests [29, 36].

Our field site, Rincón del Socorro, is a 120 km^2^ reserve within the Iberá National Park (28.6598° S, 57.4344° W), Corrientes, Argentina. It was a cattle ranch until 2002, when it was acquired by an international non-profit conservation organization, Conservation Land Trust (now called Rewilding Argentina, http://rewildingargentina.org/), and became a strict conservation area without livestock or hunting of native species [26, 29]. This site became a part of the Iberá National Park in 2018. The main terrestrial habitats are seasonally flooded grasslands, palm savannas, savannas of *Prosopis affinis* and *Vachellia caven* and hygrophilous forests [26]. Our field site is of interest because of its history of defaunation, since the Late Pleistocene and early Holocene and more recently with the European colonization, and the current efforts to restore ecosystem functioning in the area through rewilding [29].

### Reintroduced species

Four species of mammals have been reintroduced since the Rincón del Socorro reserve was created. The first species was giant anteaters (*Myrmecophaga tridactyla*) in 2007, then collared peccaries (*Pecari tajacu*) and pampas deer (*Ozotoceros bezoarticus*) in 2015, and lastly lowland tapir (*Tapirus terrestris*) in 2017. The origin of these animals varies, most come from captivity and many are rehabilitated animals that were injured or taken from the wild by poachers. The pampas deer individuals come from wild populations in the east of Iberá, where they are at high risk from hunting and dog attacks [29]. All animals went through a quarantine process, except for pampas deer because they did not came from captivity. After this, they went through an acclimation phase in the reserve where they were introduced to native food and their health status was checked. At this pre-release stage, animals were fitted with VHF transmitters. Once the pre-release period was complete, the enclosures were opened and the animals exited by themselves [29].

We have limited our study to these four species and not included any of the native species or other potential reintroductions for this site, because of the lack of wildlife tracking data.

### Framework

**Fig 1:**
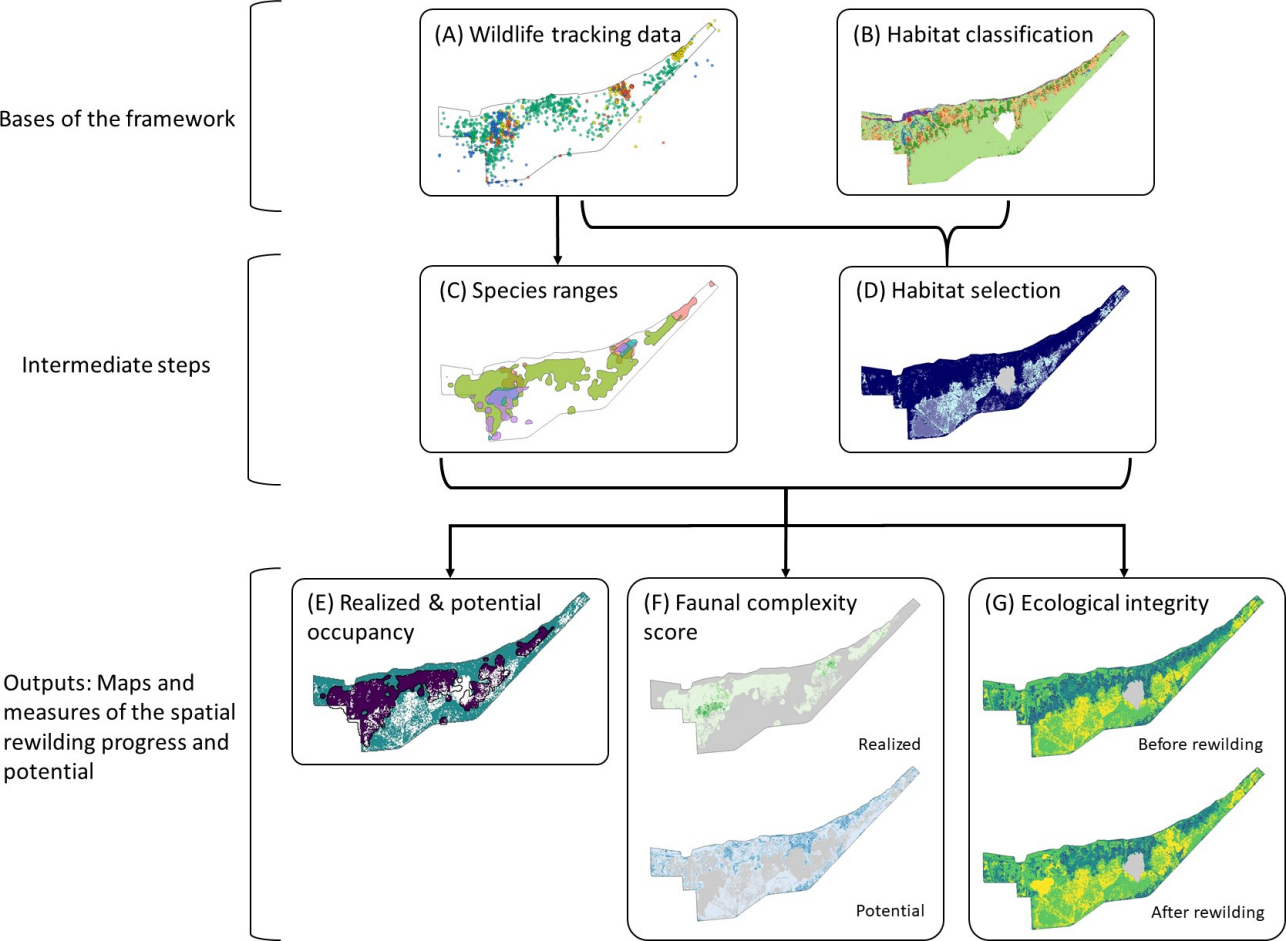
Schematic presentation of the framework workflow. The bases of this rewilding monitoring framework are (A) wildlife tracking data and (B) a habitat classification of the site. (C) The reintroduced species ranges are calculated from the wildlife tracking data. Both wildlife tracking data and the habitat classification are used to obtain (D) the habitat selection of the reintroduced species. We use the species ranges and habitat selection, to calculate and map three different and usable outputs: (E) the realized and potential occupancy, (F) the realized and potential faunal complexity, and (G) the ecological integrity before and after rewilding. These outputs show the spatial progress and potential of the rewilding project. The maps in (D) and (E) show only one of the reintroduced species maps, as an example.

The framework for spatially monitoring progress of rewilding projects has two bases: (1) habitat classification of the site, which uses first-hand knowledge of the site and satellite data, and (2) GPS locations of the reintroduced animals, through wildlife tracking data (Fig 1).

The framework consists of:

- Wildlife tracking data in the form of GPS points (Fig 1A).
- Habitat classification of the site (Fig 1B).
- Calculation of species range of the reintroduced populations from wildlife tracking data (defined as the area of occupancy) (Fig 1C).
- Habitat selection analysis based on the wildlife tracking data and habitat classification (Fig 1D).
- Identification of areas of realized and potential occupancy of reintroduced species through mapping the species range and the habitat selection (predicted probability of presence) (Fig 1E).
- Calculation and mapping of realized faunal complexity score and potential faunal complexity score. Ranging from one species to the total number of species reintroduced through the project (Fig 1F).
- Calculation and mapping of ecological integrity before and after rewilding. Ecological integrity concept based on our colleagues Torres et al. [20], but focused on changes in trophic complexity (Fig 1G).

### Data collection

We obtained GPS coordinates of occurrences for all four species recorded by rangers at Rincón del Socorro. When the animals were first reintroduced to the reserve, many were fitted with VHF transmitters. Peccaries and pampas deer form groups and only one or two individuals per group where typically collared. The offspring of the reintroduced animals, with the exception of tapirs, were not collared. The rangers located animals using a hand-held antenna during periodic sightings and status checks, and recorded spatial coordinates using a GPS device. We removed duplicated points, and points without a clear identification. We retained 252 presence points of lowland tapir from eight individuals, 188 of pampas deer from nine individuals, 886 of collared peccary from 35 individuals and 1070 of giant anteater from 25 individuals for our analyses. This data was collected from the year 2012 to 2017.

### Data analysis

#### Habitat classification

We classified the study site into ten land cover types (Fig 1B), including main habitat types for the reintroduced mammals: closed-canopy forest, free-standing trees in a grassland matrix, short grassland, tall grassland, palms canopies, *Vernonia* shrubland, aquatic vegetation, water, bare ground and artificial surfaces. Closed-canopy broadleaf forest are gallery forests surrounding water streams; they consist of trees of several species including *Sideroxylon obtusifolium*, *Eugenia uniflora*, *Scutia buxifolia Geoffroea decorticans*, *Myrcianthes pungens* [37]. The free-standing trees are individuals of *Vachellia caven* and *Prosopis affinis* surrounded by a grassland matrix, forming a savanna. Short grasslands have a high abundance of *Paspalum almum*, *Axonopus fissifolius*, and *Setaria parviflora*. Tall grasslands are dominated by *Sorghastrum setosum* and *Andropogon lateralis*. The palms are individuals of *Copernicia alba* surrounded by a grassland matrix, forming palm savannas. The shrublands are dominated by species of the *Vernonia* genus and the aquatic vegetation is made out of floating vegetation in the wetland or in small lakes. The artificial surfaces are all of the constructions and buildings, and the bare ground is mostly composed of dirt roads.

The classification was done in a two-step procedure, exploiting access to very high resolution imagery (1.2 m resolution) over part of the reserve, and extending this initial high resolution classification over the entire reserve using coarser imagery (10 m resolution).

First, we used a high-resolution (1.2 m) Worldview3 image collected on 21 July 2017 that covered 61% of the study site. In QGIS [38], we selected training pixels of known habitat type based on our field-based knowledge of the site and used them to train a neural network in R [39], which was then used to predict habitat type in the remaining pixels. The training data was split into 80% training and 20% validation subsets. Since land cover classes were not represented equally in the training data (or in the landscape), we downsampled common classes and upsampled rare classes, in order to improve the final classification. All predictor variables were centred and scaled. The model consisted of two dense layers with 100 neurons and an output layer that produces the classification using the softmax activation function. To prevent overfitting, we used a dropout rate of 0.1 between layers. The model was compiled using the sparse_categorical_crossentropy loss function and adam optimizer. The model was trained during 100 epochs with a batch size of 1000. To evaluate classification accuracy we computed overall accuracy and Cohen’s kappa score.

In the second step, we used the high-resolution land cover map to extend the land cover classification to the entire study site using Sentinel 2 imagery taken between 17 Jan 2017 and 17 Jan 2018. We obtained 12 images with low cloud cover (< 5%) over this period, and masked remaining clouds. We used 10 spectral bands (bands 2-8A, 11–12) at a resolution of 10 m. We trained a second neural network, in which the training data was taken from the WorldView3 classification. The problem is thus posed as a regression problem and the neural network predicted the proportional cover for each class, based on the 1.2 m WorldView pixels within a 10 m Sentinel 2 pixel. The training data set was split into 80% training and 20% validation subsets. All predictor variables were centered and scaled. The model consisted of two dense layers with 200 neurons and an output layer using the softmax activation function. To prevent overfitting, we used a dropout rate of 0.1 between layers. The model was compiled using the MSE (mean squared error) loss function and RMSProp optimizer. The model was trained during 200 epochs with a batch size of 10000. Model fit was computed using r-squared. Finally, we used the trained model to predict land cover across the entire reserve, using the Sentinel 2 imagery.

The neural networks were constructed using the Keras API with a Tensorflow backend, operated from R [39] using the keras package [40].

The study site contained two recently burned areas that could therefore not be classified into their actual land cover class. One burn scar could, post-hoc, confidently be attributed to one of the land cover classes (tall grassland) based on our field observations. The second burn scar was omitted from the land cover mapping and subsequent analyses, as the burned vegetation was a mosaic of different land cover classes and could thus not be attributed to a single class.

#### Habitat selection

We calculated the species ranges through Kernel Density Estimates (KDE) in R with the packages tmap [41] and tmaptools [42] (Fig 1C), for all individuals of each species with more than 10 observations, we excluded all points outside of the reserve and established a 95% volume contour [43]. We generated random available points to create pseudo-absences [44]. To do this, we created a circumference with an area equal to the median species range, surrounding all presence points, the pseudo absences points were generated outside these areas. We did this to take into account possible spatial sampling bias of the radio tracking that constitute the presence points. The number of pseudo-absence points for each species was the same as the presence points [44, 45]. For all presence and pseudo-absence points, we extracted the habitat proportion values out of a buffer area surrounding the point corresponding to the median range size of each species. These buffers were the same size as the previously mentioned circumferences used in the creation of pseudo-absences. We joined the presence and pseudo-absence points, extracted habitat proportion values into one dataset and fitted a random forest model [46] from package party [44, 47]. We did this to model the habitat selection (or predicted probability of presence) of the species based on the proportion of the four main habitat types: broadleaf forest, free standing trees, short grassland and tall grassland, we fitted one model per reintroduced species (Fig 1D).

We aggregated the raster with classified habitats into 30 m by 30 m cells, through the aggregate function from the raster package [48], calculating the mean proportion of each habitat category in the cell. We chose this size, instead of the original 10 meter resolution, to account for the errors in GPS measurements. We used the predict function of the raster package [48] to predict the probability of presence of each species onto the aggregated raster of our site. To evaluate the performance of the models we calculated the AUC [49] with the function roc and auc of package pROC [50].

#### Rewilding occupancy and ecological integrity measures

We identified areas of realized and potential occupancy for each species within the site (Fig 1E). Areas of realized occupancy consisted of areas with predicted probability of presence of more than 0.5 that were within the species range. Areas of potential occupancy were areas with predicted probability of presence of more than 0.5, that were outside of the species range. We calculated a faunal complexity score (Fig 1F), based on the number of reintroduced species that occupy (realized faunal complexity score) or are predicted to occupy (potential faunal complexity score) an area. For our case study, this score ranged from one species to four species. To calculate the scores we used the functions st_intersection and st_difference from the package sf [51] to measure the overlap and difference between the predicted probability of presence and the range of each species.

We calculated the ecological integrity scores as the realization of the rewilding potential for each raster cell within our site (Fig 1G). We based this on the concept from our colleagues Torres et al. [20], they presented three guiding principles for ecological integrity: the naturalness of disturbances, the connectivity of terrestrial and aquatic systems, and the composition and complexity of the trophic network. We evaluated a subset of these guiding principles considered by Torres et al. [20]. We focused on the changes in the trophic network in order to narrow down our assessment to the aspect that is being actively restored and can be spatially evaluated. The ecological integrity score ranges from 0 to 1, a low ecological integrity means that the rewilding potential for that area was not realized, and a high ecological integrity means that the area is occupied by the species predicted to be there. We assigned a value of zero to a cell when we predicted it to be occupied by the species but it was outside of the species range. We assigned a value of one to a cell when we predicted it to be occupied by the species and it was within the species range. We also assigned a value of one when the species was not predicted to occupy the cell, whether this was or not within the species range, since the species range can include areas of passage and are not suitable for the species. The reason why cells where the species is not predicted to occur are assigned a high ecological integrity is that these cells are already fulfilling their rewilding potential. These values were recorded for all species, summed and divided by the total number of species to create the ecological integrity score.

All statistical analyses were performed using R [39], and in addition to the packages mentioned previously, we used ggspatial [52], ggplot2 [53], stars [54], tidyverse [55], viridis [56].

## Results

**Fig 2:**
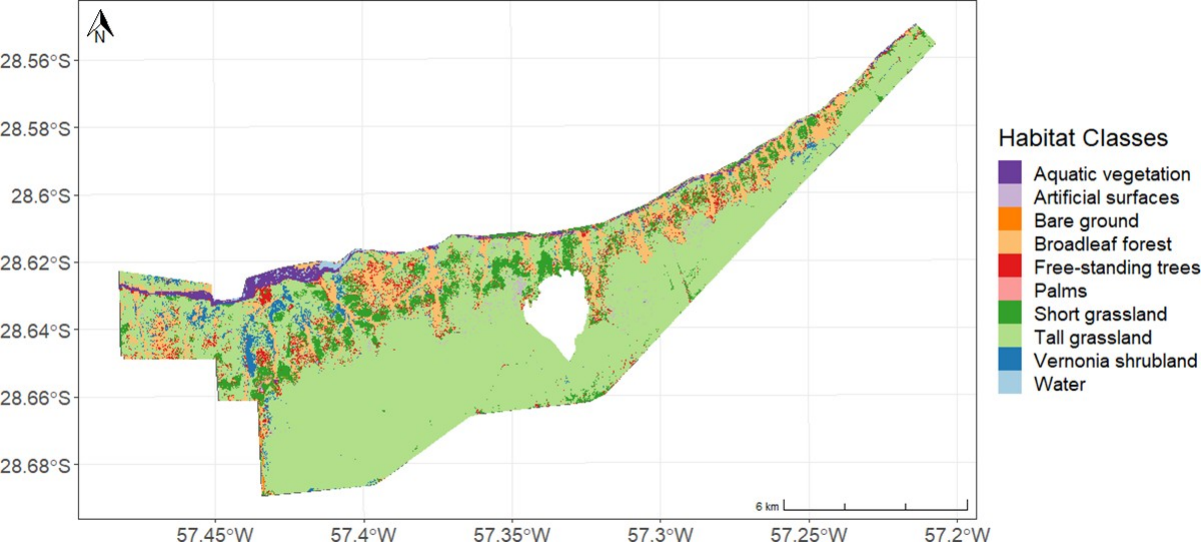
Habitat classification. Map of habitat classes of the study site, Rincón del Socorro. The site was classified into ten habitat classes: aquatic vegetation, artificial surfaces, bare ground, broadleaf forest, free-standing trees, palms, short grassland, tall grassland, *Vernonia* shrubland, and water.

The majority of our field site is occupied by tall grasslands (70%), followed by closed-canopy broadleaf forest (11%) and short grasslands (9%) (Table 1, Fig 2). The northern half of the site is a heterogeneous landscape that borders with the main wetland to the north (shown in our classification as areas with aquatic vegetation and water in the very north of the site). The northern half of the site is a mosaic of closed-canopy broadleaf forest, short grassland, free-standing trees in a grassland matrix, palms in a grassland matrix, and *Vernonia* shrubland. The southern half of the site is dominated by tall grassland.

**Table 1:** Area estimations per habitat class.

Habitat class	Area estimations (km^2^)
Aquatic vegetation	2.42 km^2^
Artificial surfaces	0.89 km^2^
Bare ground	0.14 km^2^
Broadleaf forest	12.46 km^2^
Free-standing trees	4.47 km^2^
Palms	0.19 km^2^
Short grassland	10.05 km^2^
Tall grassland	81.31 km^2^
*Vernonia* shrubland	3.29 km^2^
Water	0.45 km^2^

Area estimate in km^2^ for each of the ten habitat classes in the study site. Estimation obtained from the habitat classification of the site.

The two-step habitat classification procedure yielded reliable models. The overall accuracy of the first step (based on the 1.2 m WorldView3 image) as calculated on the 20% hold-out test data was 0.97, with a kappa score of 0.92. The overall fit of the second model (based on the 10 m Sentinel 2 imagery) as calculated on the 20% hold-out test data was r^2^ 0.93. The class-specific r^2^ values ranged from 0.32 for palms to 0.98 for the burned area.

**Fig 3:**
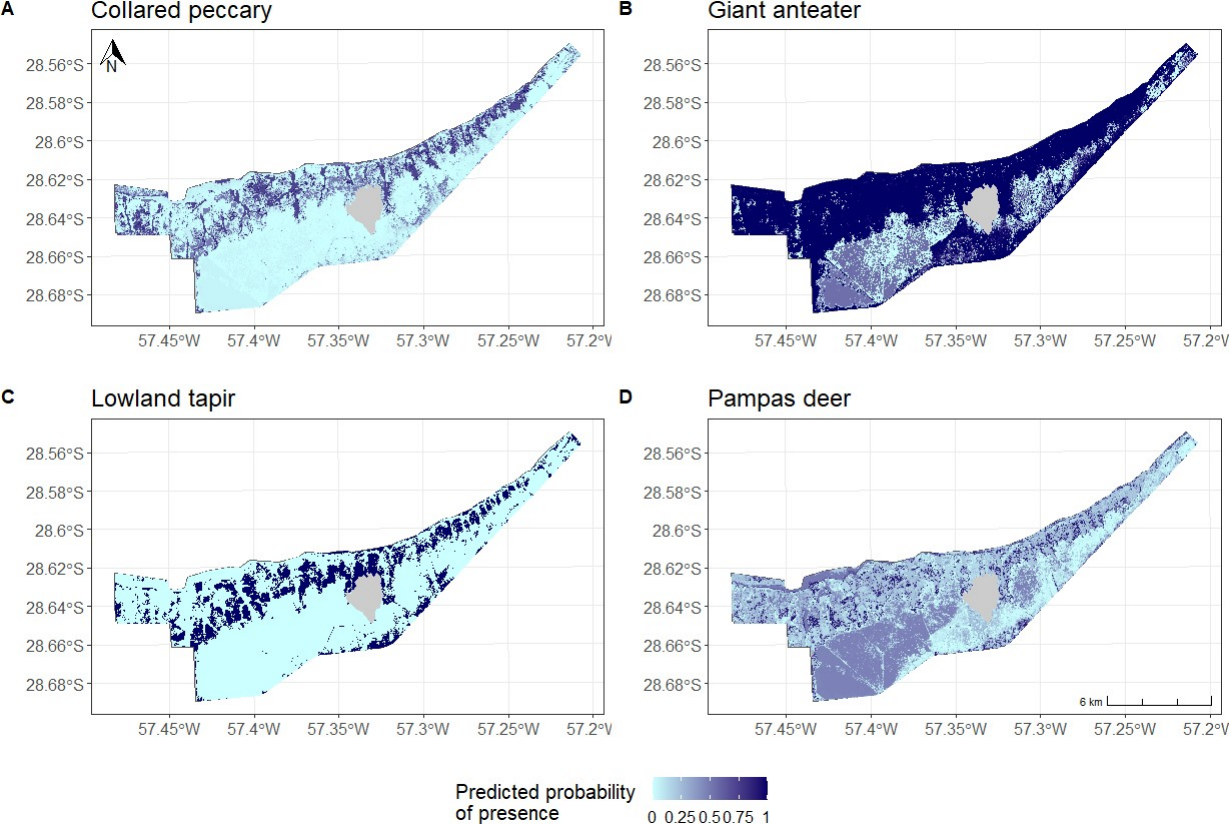
Habitat selection of reintroduced species (predicted probability of presence). Maps of predicted probability of presence for the four reintroduced species, giant anteater, pampas deer, lowland tapir and collared peccary, with a gradient from 1 (highest probability of presence) to 0 (lowest probability of presence).

We modelled the predicted probability of presence of collared peccary, giant anteater, lowland tapir and pampas deer, based on the habitat classification of the site (Fig 3). The model predicted collared peccary (AUC = 0.97) to occupy broadleaf forest areas. Giant anteater (AUC = 0.99) was predicted to be present in most of the north of the site, which is characterized by a mosaic of forests, short and tall grasslands, free-standing tree and palm savannas. Lowland tapir (AUC = 0.95) was predicted to mostly occupy savanna areas adjacent to the broadleaf forests. Pampas deer (AUC = 0.92) was predicted to occupy short grasslands, and free-standing tree and palm savannas (Fig 3). All four species are predicted to be mostly absent from the homogeneous tall grasslands in the southern half of the site.

**Fig 4:**
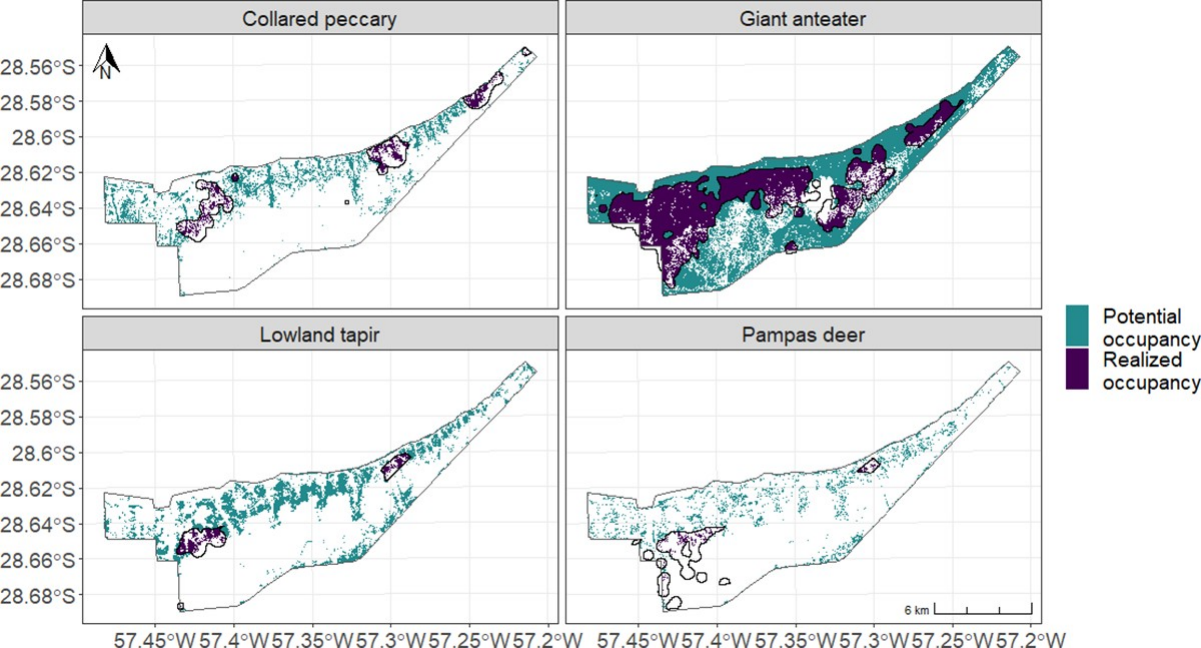
Realized and potential occupancy of reintroduced species. Predicted probability of presence inside and beyond current species range for giant anteater, pampas deer, lowland tapir and collared peccary, in our study site. Realized occupancy of reintroduced species was defined as predicted probability of presence of more than 0.5 inside the current species range, and potential occupancy of reintroduced species was defined as predicted probability of presence of more than 0.5 beyond the current species range. The areas delimitated in black are the species ranges of each species.

The species with the biggest area of realized and potential occupancy is the giant anteater (Fig 4, Table 2). This species already occupies 42.9% of the area predicted to be selected by the species (Table 2), it is the only species with a high percentage of realized occupancy of reintroduced species. Peccary, tapir and pampas deer have so far only occupied 22.9%, 10.5% and 10.0% of their predicted selected areas, respectively (Table 2). These three species have occupied small, isolated areas within their predicted distribution in the site (Fig 4).

**Table 2:** Area and proportion of realized and potential occupancy of reintroduced species.

	Realized occupancy of reintroduced species		Potential occupancy of reintroduced species	
**Peccary**	4.37 km^2^	22.9%	14.70 km^2^	77.1%
**Giant anteater**	40.06 km^2^	42.9%	53.29 km^2^	57.1%
**Tapir**	2.58 km^2^	10.5%	21.88 km^2^	89.5%
**Pampas deer**	1.11 km^2^	10.0%	10.02 km^2^	90.0%

Total area of realized occupancy (predicted probability of presence of more than 0.5, inside the species range) per species and proportion of realized occupancy of the total area with predicted probability of presence of more than 0.5 for that species. Total area of potential occupancy (predicted probability of presence of more than 0.5, beyond the species range) per species and proportion of potential occupancy of the total area with predicted probability of presence of more than 0.5 for that species.

**Fig 5:**
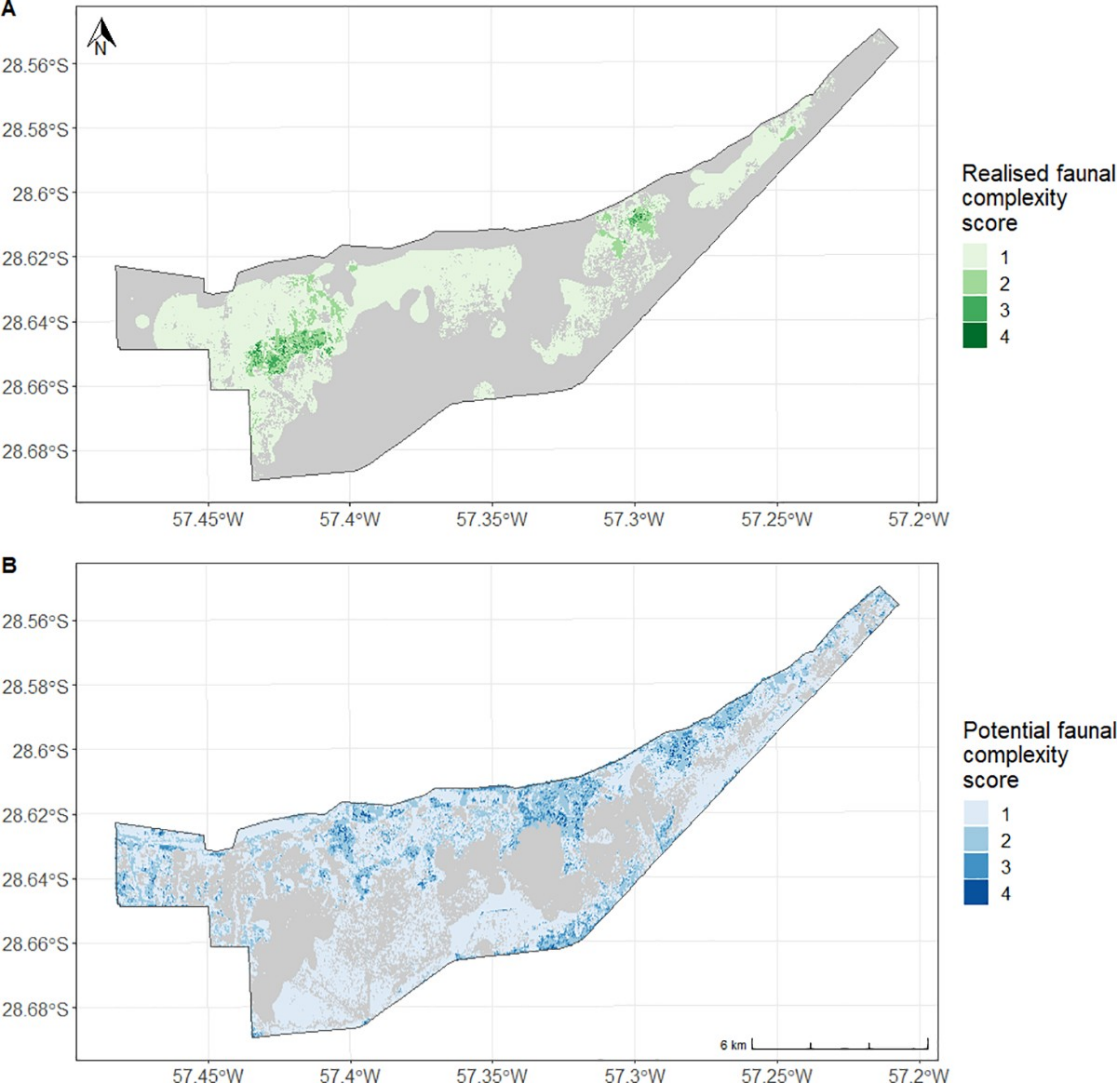
Faunal complexity score. (A) Realized faunal complexity score. Map of the number of species with realized faunal complexity (predicted probability of presence of more than 0.5, inside the species range) in our site. (B) Potential faunal complexity score. Map of the number of species with potential faunal complexity (predicted probability of presence of more than 0.5, beyond the species range) in our site.

We calculated faunal complexity as the predicted occupancy for all four species overlapped, this faunal complexity could be realized or potential. There are three areas of high realized faunal complexity in the site, to the left, center-right and right of the north of the site, where the reintroduced species are occupying their predicted ranges (Fig 5A). The area currently occupied by all four mammal species is small (0.07 km^2^) and gets progressively larger as the number of overlapping species decreases (37.96 km^2^ for one species), this pattern is consistent for both realized and potential rewilding (Table 3). Much of the area with high potential faunal complexity is found between the clusters of high realized faunal complexity, pointing at the presence of a general area of high predicted occupancy at the north of our site (Fig 5B). The majority of the area with realized and potential faunal complexity has a low score, 33% and 43.6% respectively.

**Table 3:** Realized and potential faunal complexity score.

Faunal complexity score	Realized faunal complexity (km^2^)	% of total predicted faunal complexity	Potential faunal complexity (km^2^)	% of total predicted faunal complexity
**1 species**	37.96 km^2^	33%	50.18 km^2^	43.6%
**2 species**	3.79 km^2^	3.3%	17.53 km^2^	15.3%
**3 species**	0.74 km^2^	0.6%	4.22 km^2^	3.7%
**4 species**	0.07 km^2^	0.1%	0.49 km^2^	0.4%
**Total**	42.56 km^2^	37%	72.42 km^2^	63%

Total area of realized faunal complexity (predicted probability of presence of more than 0.5, inside the species range) from 1 to 4 species. Total area of potential faunal complexity (predicted probability of presence of more than 0.5, beyond the species range) from 1 to 4 species. The percentages represent the proportion of the total site with that specific faunal complexity score, whether realized or potential. The total is 63% because the remaining 37% of the site has a faunal complexity score of zero.

**Fig 6:**
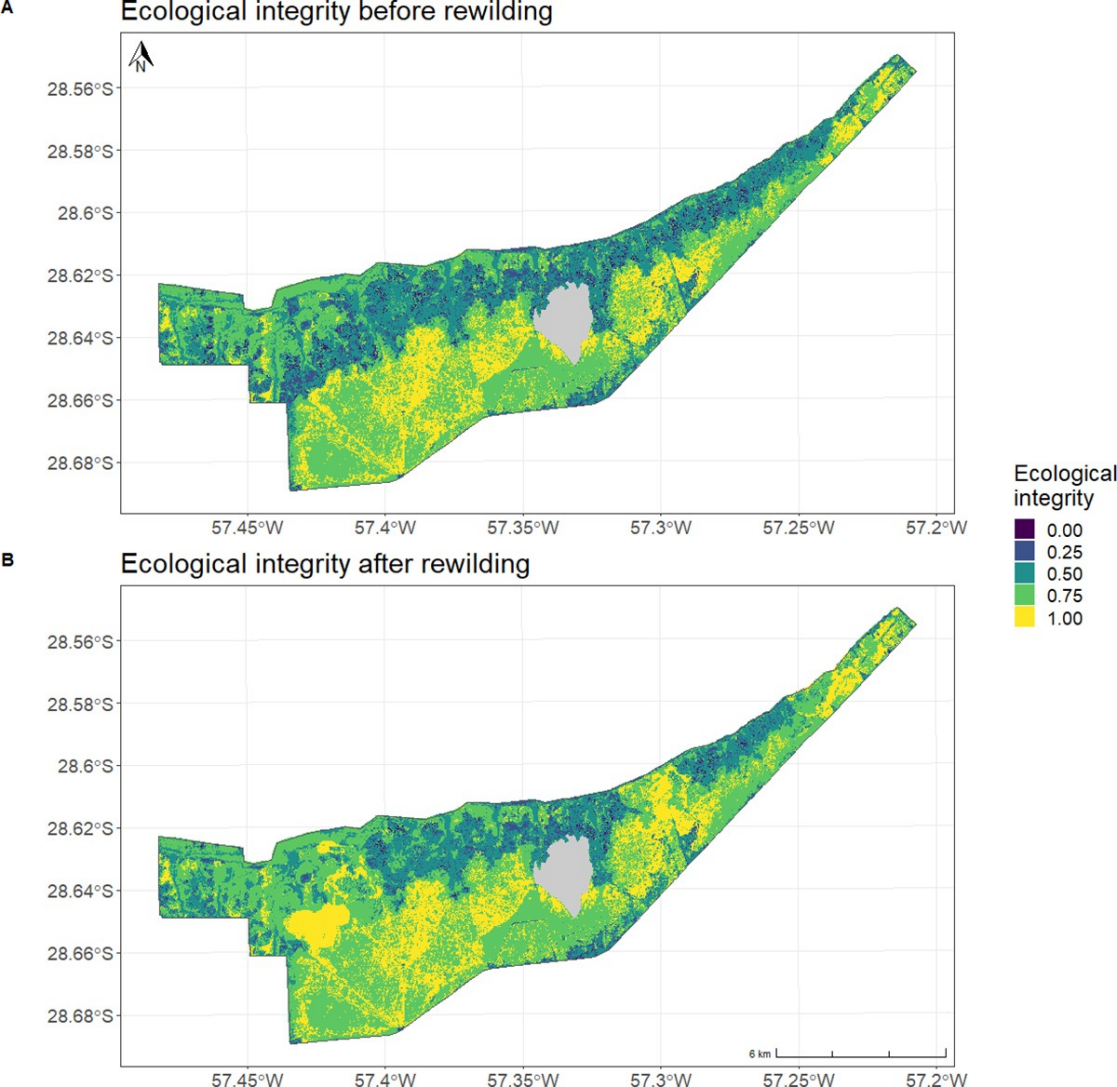
Ecological integrity. Map of magnitude of realization of the potential occupancy per cell. An ecological integrity of zero represents no occupancy of any of the species predicted to be there. An ecological integrity of one represents the realization of all predictions for that cell, including species occupancy and species absence. Before (A) and after (B) rewilding.

Rewilding had a positive effect on ecological integrity and resulted in an overall increase of areas with higher scores (Fig 6, Table 4). Before rewilding there was low ecological integrity in the northern half of the site, an area characterized by a heterogeneous mosaic of forests, grasslands, and free-standing trees and palm savannas, and high ecological integrity in the southern half, dominated by tall grassland and not predicted to be occupied by most species considered in this case study (Fig 6). There are three clear areas where the rewilding provided an increase in ecological integrity within the site compared to before the rewilding (Fig 6). These areas are in the northern part of the site, which had the most potential to increase its ecological integrity score, and predictively correspond with the areas with high realized faunal complexity (Fig 5A).

**Table 4:** Ecological integrity score.

Ecological integrity score	Area before rewilding (km^2^)	% of the site before rewilding	Area after rewilding (km^2^)	% of the site after rewilding
**0**	1.51 km^2^	1.3%	0.47 km^2^	0.4%
**0.25**	8.99 km^2^	7.7%	5.74 km^2^	4.9%
**0.5**	30.88 km^2^	26.4%	24.47 km^2^	20.9%
**0.75**	53.26 km^2^	45.5%	58.68 km^2^	50.1%
**1**	22.39 km^2^	19.1%	27.67 km^2^	23.7%

Total area of the site with different levels of ecological integrity before and after rewilding. Ecological integrity varies from 0 to 1, a score of zero represents no occupancy of any of the species predicted to be there. On the other end of the spectrum an ecological integrity of one represents the realization of all predictions for that cell, including species occupancy and species absence. The percentage represents the proportion of the site occupied by the ecological integrity score either before or after rewilding.

The area covered by ecological integrity scores lower or equal to 0.5 decreased with the incorporation of rewilding, and the area with scores higher than 0.5 increased (Table 4). After rewilding 73.8% of the site had a high ecological integrity (score higher than 0.5), instead of the previous 64.6% (Table 4).

## Discussion

Through our framework we were able to assess the spatial reversal of defaunation through rewilding of our case site and the remaining occupancy potential of the reintroduced species. We found that 37% of the areas predicted to be occupied by the reintroduced species were realized, leaving 63% of that area with potential to have increased faunal complexity through the rewilding efforts, illustrating time lags in population build-up and local range expansions. We could also show that the ecological integrity of the site increased with rewilding.

In the case study we only considered the four reintroduced species, all of which we have wildlife tracking data for. Our predicted, realized and potential occupancies are from these species, but other potential rewilding species could have been included in the models if we had the tracking data to analyze. It may also be relevant to use parts of this framework to study and assess the condition of native populations in cases where tracking data is available, such as capybaras (*Hydrochoerus hydrochaeris*) and marsh deer (*Blastocerus dichotomus*) [34, 57].

In our case study in Argentina, we found that the four reintroduced species had realized from 10% to 43% of their predicted range. The giant anteaters had the biggest area predicted to be occupied and realized the largest proportion of it. Giant anteaters have been reintroduced in the site the longest (first release in 2007) and have one of the highest number of individuals. In a previous study comparing species range and habitat selection of giant anteaters in Iberá and Pantanal, Di Blanco et al. [43] suggested that the reintroduced populations may not have reached carrying capacity and have lower level of social interaction, and consequently having larger species ranges. The results of our implementation of the framework showed that the reintroduced species have not reached their rewilding potential. The time since the first releases of reintroduced individuals could have influenced this progress [16]. Giant anteaters have been in the site for ten years and have occupied the largest proportion of their predicted selected areas, whereas the other three species have been present for two years or less and have the majority of their predicted selected areas left to fill. The reintroduced populations in our case study have not been in the site for long, and are likely to be far from equilibrium and not occupy all suitable habitats. This may lead to habitat truncation and an underestimation of the species potential distribution [58]. Given the small size of the field site (120 km^2^), its mosaic nature, and the high mobility of all of the species, we consider that all species, regardless of the time since release, had the possibility to access the different habitat types available in the site and select their preferred ones.

The ecological integrity maps show the spatial progress of rewilding. The area with high ecological integrity (score higher than 0.5) increased with rewilding. From all of the pressures and state variables used in the original characterization of ecological integrity from Torres et al. [20], we chose to narrow down our assessment to terrestrial large-bodied fauna. There are other relevant contributors to ecological integrity such as how natural the fire and hydrological regimes are, and how fragmented the landscape is, which could also be potentially studied through remote sensing. In our case, the landscape had low fragmentation and some of the fire and water dynamics were driven by human intervention. For our purposes, the guiding principles from Torres et al. [20] related to these variables, which are naturalness of disturbances and connectivity of the terrestrial and aquatic systems, were not relevant or could not be quantified at this point. However, applying their framework and considering a broader set of dimensions such as fire and hydrology would provide a well-rounded understanding of the progress and dynamics in a rewilding site, even though it does not have the spatial perspective our approach does.

The majority (55%) of our site is tall grasslands which has a low potential faunal complexity score. Consequently, the habitat type that covers the majority of the site is not predicted to be occupied by the species considered in this project. In other sites, pampas deer have been found to select for tall grasslands [59], the lack of selection for this habitat in our study could reflect niche truncation. In our site, tall grasslands are managed through controlled burns. The fragmentation of the surrounding landscape has led to the loss of natural fire spread and controlled burns are used to avoid the excessive accumulation of dead biomass that could fuel very hot and destructive wildfires [60]. Due to this being a rewilding site another option to manage this area would be to consider the reintroduction of other herbivore species that could utilize this habitat. A potential species to reintroduce for this purpose is the horse (*Equus ferus*), South America used to have its own species of horses including horses that were either very closely related to or conspecific with extant horses that became extinct in the Pleistocene [61]. Horses can consume the less palatable tall grasses and promote heterogeneity in this very homogeneous part of the reserve [62].

This framework was prepared for large terrestrial animals and through the case study has been proven to work for this group. Other groups may present challenges that necessitate adjustments. Bird species can also be subject of reintroductions, but are able to fly to other sites and natural areas, which may mean that areas where the individual only overflew would have to be disregarded and movement outside of the reserve might have to be considered. This framework can also be used to monitor passive rewilding and the introduction of functional analogues [2]. We do not include a time scale in this case study because the individuals were taken off radio collars after some time, by keeping the data from all years together we get a more realistic analysis, but lose the possibility of doing a year by year range filling of the site. This can also be a useful strategy for rewilding projects that are young or have few individuals of each species, this way they can maximize the available data.

Remote sensing is a useful and versatile tool, that has been used for diverse purposes such as to study land-use and land-cover change, and to identify suitable habitats for species [63]. Even though our framework does not provide direct proof on ecological function filling, there is potential to use remote sensing also to study the ecosystem effects of wildlife reintroductions spatially, for instance by researching changes in fire regime and shifts in habitat [19].

Our framework has real-life applications. Through it, practitioners can learn which areas of the site are occupied by the reintroduced fauna, which are predicted to be occupied but have not, and which are not predicted to be occupied by the currently reintroduced species. We do not consider that having a high faunal complexity across the site should be a goal, since not all areas will be suitable for the reintroduced species. Instead, the focus should be on achieving a high ecological integrity, in other words, the realization of potential occupancy. The results of applying our framework can help practitioners decide where to perform future releases of individuals and assess if the site could benefit from further reintroductions, in order to achieve their restoration goals which are often open-ended and focused on ecological processes [17]. The habitat selection analysis presented in this study is not something many practitioners are accustomed to and may be an obstacle to implementation. High-resolution satellite images, such as the one used in our habitat classification, are expensive and can be financially out of reach. Wildlife tracking technology can also be prohibitively expensive, especially with the need to acquire many devices to track a big number of individuals. Fortunately, high-resolution satellite imagery and wildlife tracking technology are becoming increasingly cheap, accessible and high quality [64–67]. Technological developments are facilitating an increasingly high spatio-temporal resolution to our combined wildlife tracking-remote sensing approach to monitor rewilding progress.

Our framework provides spatial understanding of rewilding progress and potential, responding to the need for more integration between rewilding science and practice [7, 10, 27]. The application of this framework will provide guidance to practitioners and decision makers when pursuing rewilding projects and managing their progress towards restoration goals. Measuring and interpreting rewilding progress allows us to learn how to better apply this restoration strategy and reverse part of the defaunation that our ecosystems have and are currently experiencing.

## Supporting information

S1 Appendix Wildlife tracking data. (CSV)

S2 Appendix Classified habitats file. (TIF)
